# Tale of a Blocked Chest Drain Resulting in Tension Pneumothorax: Are We Always Competent Enough to Troubleshoot a Chest Drain?

**DOI:** 10.7759/cureus.66037

**Published:** 2024-08-02

**Authors:** Mohammed Adul Hai Amer, Saquib Siddiqui

**Affiliations:** 1 Respiratory Medicine, Freeman Hospital, Newcastle Upon Tyne, GBR

**Keywords:** blocked chest drain, respiratory disease, chest drain troubleshooting, chest drain, pneumothorax ptx

## Abstract

Intercostal chest drain (ICD) insertion for pleural effusion or pneumothorax is one of the most routinely performed procedures. Patients with ICDs usually remain in a respiratory, medical, or cardiothoracic ward or a critical care unit. Despite it being a common procedure, there seems to be limited competency in troubleshooting chest drain when it is not functioning. From our daily experience working with junior doctors and doctors from specialties other than respiratory medicine, we feel there is a significant lack of training and troubleshooting skills to handle a non-functioning chest drain. This is a matter of grave concern, as inappropriate assessment can lead to the insertion of additional chest drains.

In the current practice in the United Kingdom, we do not have out-of-hours respiratory cover, particularly in the district general hospitals, and the on-call medical team will not always have a registrar or any other team member with respiratory work experience. Therefore, we often fall short of managing ICD-related complications which results in the incorrect assessment of the patient's condition further leading to incorrect action plans, causing more harm to the patient.

We present one such case, where a young pregnant lady in the respiratory ward who underwent thoracostomy with an ICD placement developed a near-tension pneumothorax secondary to block in the drain. The medical team's inexperience in troubleshooting led to a wrong treatment plan for the patient. Although ICD-related complications are frequent, very few reviews and guidelines are available in the literature which covers it comprehensively. Therefore, we aim to enlighten this less-explored area of clinical practice along with a review of literature.

## Introduction

Intercostal chest drain (ICD) insertion for pleural effusion or pneumothorax is one of the most routinely performed procedures. Although commonly performed, there seems to be limited competency in troubleshooting chest drain when it is not functioning. It is more frequently observed among medical doctors with no prior experience in respiratory medicine. There is a clear lack of training and troubleshooting skills to handle a non-functioning chest drain. This is a matter of grave concern, as inappropriate assessment can lead to the insertion of additional chest drains. Therefore, we aim to enlighten this less-explored area of clinical practice along with a review of literature.

## Case presentation

**Figure 1:**
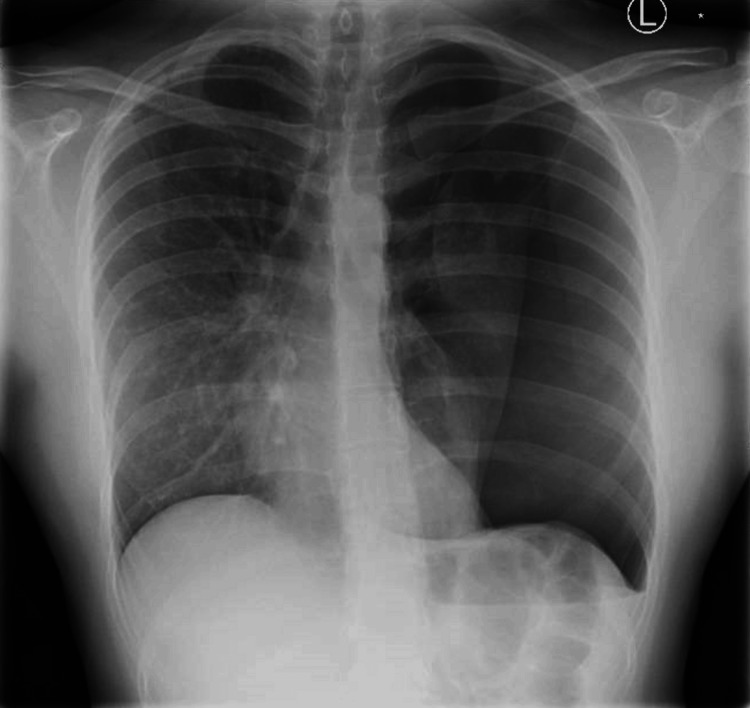
Large left-sided pneumothorax

**Figure 2:**
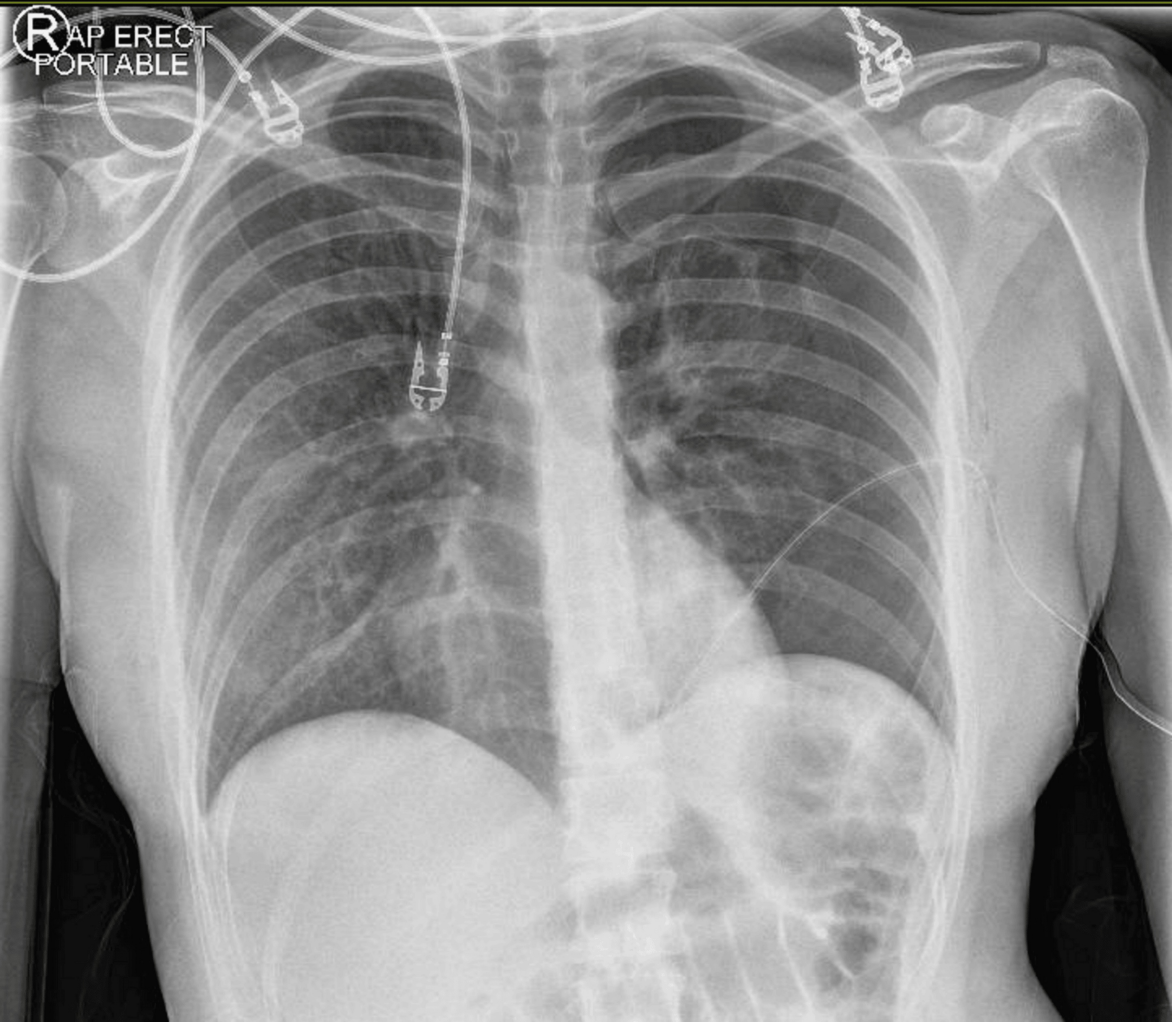
Post-ICD insertion status. Partial resolution of the pneumothorax ICD: intercostal chest drain

A young lady in her 20s, with a gestational age of six weeks, presented with shortness of breath and left-sided chest pain. On examination, she was tachycardic, tachypnoeic, and desaturating with reduced air entry on her left side. Therefore, she was started on supplementary oxygen of 15L with a non-rebreathing face mask. An urgent chest X-ray was done, and it revealed a large left-sided pneumothorax (Figure 1). Consequently, she underwent a left-sided thoracostomy, and a 12-French ICD was inserted via Seldinger's technique. A repeat X-ray showed a partial resolution of the pneumothorax (Figure 2). 

The patient remained hemodynamically stable for the next 48 hours, not requiring any oxygen support. Later, at night, she started feeling breathless with heaviness in her chest. On inspection, the ICD was found neither swinging nor bubbling. A chest X-ray showed a worsening of the pneumothorax. The junior doctor covering the medical wards decided to put the ICD into suction, and it started to bubble again. However, the doctor did not check if it was swinging. Despite a couple of hours of suction, her condition did not improve. Therefore, it was escalated to the on-call senior medical registrar. On his arrival, he noticed the ICD was only bubbling in the water seal bottle and not swinging, likely due to suction rather than a patent ICD. So, he stopped the suction and started troubleshooting. On repeating the X-ray, the ICD appeared to be within the pleural cavity, in position, and not kinked, although placed slightly deeper. Then he proceeded to investigate for drain patency. On flushing the drain tube with 10 ml of normal saline, it appeared alright from the three-way tap towards the drainage system but showed a slightly slower flow towards the patient. Therefore, the dressing was removed, and the drain tube was noted to be attached too tight, to the extent of causing a mild compression effect from the dressing itself. The patient was given a new dressing that was less tightly bound, and then the drain tube started to swing and bubble instantly. A subsequent X-ray showed improvement of the pneumothorax.

**Figure 3:**
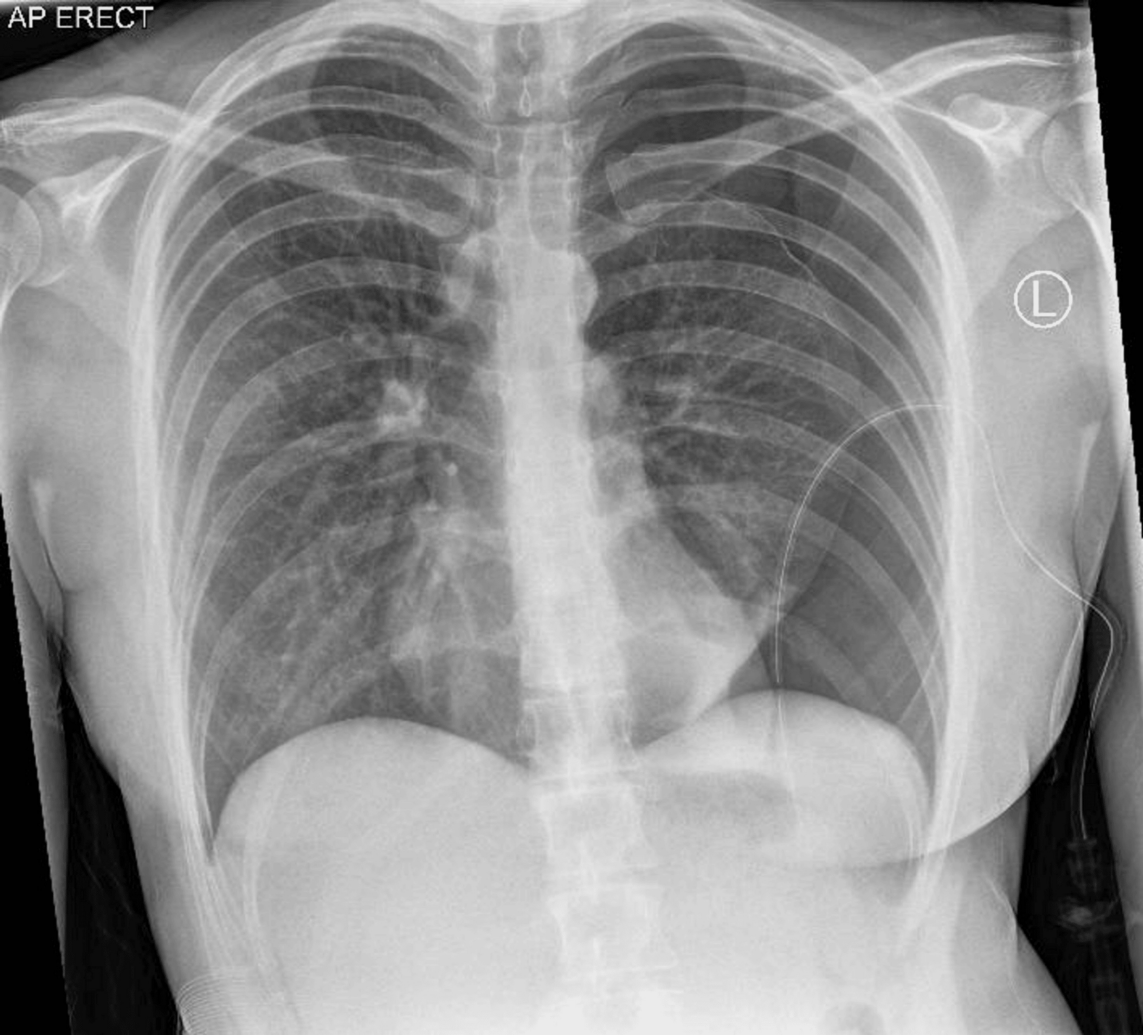
Large left-sided pneumothorax with deeply situated ICD ICD: intercostal chest drain

The patient remained stable the following day but again became unwell overnight. This time, however, she was on the brink of developing a tension pneumothorax. She required oxygen support (she had been off oxygen for the preceding three days since the ICD insertion), had a tracheal deviation to the right, and had low blood pressure (95/66 mm Hg). On inspection, the junior doctor found that her ICD was not swinging and bubbling again, so she was put back on suction and an urgent chest X-ray was organized. It showed a worsening of the pneumothorax with tracheal deviation to the right, and the ICD was deep within the pleural cavity (Figure 3).

Later that night, the case was discussed with the cardiothoracic team, and they considered the patient a candidate for a video-assisted thoracic surgery (VATS) procedure, considering the developing pneumothorax in the context of pregnancy. However, she was not stable enough for transfer or any surgical intervention immediately, so it was advised to insert a new, larger ICD. The on-call medical registrar decided to discuss the case with the critical care team for a possible transfer to the critical care unit for closer monitoring and the insertion of a larger ICD.

**Figure 4:**
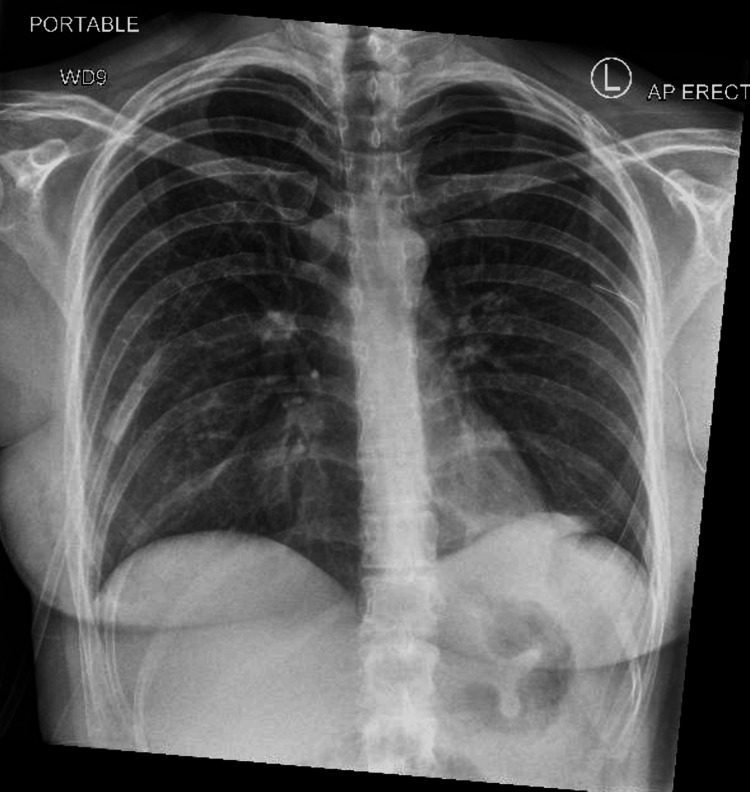
Significant improvement of the left-sided pneumothorax. ICD less deep (retracted) ICD: intercostal chest drain

Until then, no one from the medical on-call team had assessed if there was any problem with the ICD itself that could be resolved to avoid a new ICD insertion overnight. The critical care registrar on duty, who was a respiratory trainee doing his critical care rotation, decided to review the patient in the ward first to troubleshoot the ICD. On review, it was the same story: the ICD was only bubbling on suction and not swinging. On stopping suction, it was neither swinging nor bubbling. The X-ray again showed the ICD was within the pleural cavity, not kinked but very deep-seated. He decided to open the dressing and retract the tube by 2 cm. Upon opening the dressing, the ICD was noted to be very deeply inserted as no length markings were visible. After retracting the ICD by 2 cm, the portion of the tube that had been inside the chest wall earlier was found to be full of debris. It was cleaned via suctioning with a syringe through the three-way tap. The ICD was then sutured again and kept at a 12 cm length position. Dressing was applied. On opening the three-way tap, it was found to be swinging and bubbling again. The ICD was kept on suction at -1.0 kPa. A repeat X-ray after an hour showed a significant improvement of the left-sided pneumothorax and resolution of the tension features, such as the trachea returning to a central position (Figure 4). A further X-ray 24 hours later showed the resolution of the left-sided pneumothorax. Eventually, the ICD was removed, and the patient was transferred to the cardiothoracic unit for the VATS procedure.

## Discussion

Appropriate troubleshooting should be the first and foremost assessment when facing difficulties in ICD management. We often fail to address the basics and instead aim for more invasive procedures that could be easily avoided if proper troubleshooting were done initially. In our case, it saved the patient from undergoing a larger ICD insertion overnight. This is a common scenario in many respiratory or medical wards, as a significant number of patients stay in the hospital with chest drains for various reasons. Issues such as a drain losing its patency due to blockage, the drain tube being kinked on a chest X-ray, or a drain coming out of the pleural space need to be carefully assessed before deciding on further drain insertion. The literature also provided limited information about troubleshooting an ICD placed in the pleural space.

Normal physiology suggests the presence of negative intrathoracic pressure of approximately -8 cm of water [1], which allows the drain content to be drawn back towards the patient, while it reverses during expiration. Therefore, the water column in the drainage bottle should move with the respiratory cycle. This movement is called the "swinging" of the chest drain. If the drain has been inserted to treat a pneumothorax (the presence of air within the pleural space), in addition to the swinging movement, the drain will initially bubble spontaneously as the raised intrathoracic pressure forces air removal from the pleural space. Once the air starts to move out and this pressure reduces, the drain stops bubbling spontaneously but continues to bubble during coughing. Eventually, once the pneumothorax resolves, bubbling stops altogether, leaving only the swinging movement of the drain.

Common problems

So now the question is as follows: What are the common problems that can occur? We provide a summary of the common problems we face with chest drains along with their solutions.

Problem 1

The patient is coughing, is more breathless, and has started to develop chest discomfort or pain. 

Reason: This is usually indicative of re-expansion pulmonary edema (RPO). It occurs when draining large effusions too quickly or when the patient has had a pneumothorax for a while. It is caused by the rapid re-expansion of lung parenchyma associated with the restoration of normal intrapulmonary blood flow within previously compressed vessels, which causes a sudden rise in pressure and rupture of the blood vessels.

Solution: If the ICD was inserted for effusion, it is recommended to clamp the ICD and perform an immediate X-ray to confirm the diagnosis. If the ICD was inserted for a pneumothorax, it is advised not to clamp the drain and to perform an X-ray straight away to avoid developing a tension pneumothorax. In the case of pleural effusion, it usually resolves once the ICD is clamped and the patient is given a break from drainage. No clear time frame has been advised, so it will be more of a clinical decision based on patient symptoms and imaging. It gets more challenging in the case of a pneumothorax as clamping the drain will put the patient at risk of developing tension pneumothorax. Therefore, the most recent British Thoracic Society (BTS) guidance [2] recommends critical care observation, ensuring adequate oxygenation with continuous positive airway pressure (CPAP)/high-flow nasal oxygen (HFNO)/high-flow face mask (HFFM). The use of opiates and diuretics is also suggested by the BTS guidelines [2].

Problem 2

The patient is starting to develop neck pain or stiffness associated with neck/chest swelling, with or without swelling of the face (including eyelids), limbs, or abdomen.

Reason: Surgical emphysema.

Solution: Urgent imaging is needed to confirm the diagnosis. If the drain appears to be kinked on the X-ray, it can be retracted a few centimeters to aid in achieving drain patency [3,4]. If the drain's side holes seem to be out of the pleural space, a new ICD insertion and removal of the older one are needed [3,4]. If the drain positioning is correct, check the drain patency. Look for any blockages (is the drain swinging or bubbling?). If the drain is not swinging/bubbling, it can be flushed with normal saline (10-20 ml) via a three-way tap both towards the bottle and towards the patient separately to check which end is problematic. If it remains non-functional despite flushing, check the dressing, as it can often be too tight. If no issues are found, and the drain is not patent and functioning, a new ICD is needed. Lastly, if surgical emphysema develops despite a patent/functioning ICD with appropriate placement on imaging, the ICD can be put on suction or replaced with a larger-bore chest drain. If all measures fail, consult the cardiothoracic team or consider less invasive interventions such as a subcutaneous drain or skin incision after discussion with a senior respiratory physician and cardiothoracic surgeons [2].

Problem 3

The drain has stopped swinging or bubbling.

Reason: If the drain is not swinging or bubbling, it is either blocked or displaced. An ICD can become blocked due to the accumulation of blood, clots, fibrin, or debris. Tight pressure from the dressing bandages or drainage holes occluded by the chest wall can also result in a blocked drain. Otherwise, a drain can be kinked, which is easily noticed on the chest X-ray. A drain can be displaced, resulting in a non-functional drain.

Solution: Perform an immediate chest X-ray. If the tube appears to be kinked, it can be retracted a few centimeters to relieve and regain patency. If the drain is too deeply seated, it can be retracted a few centimeters to relieve pressure [3]. If the side holes appear to be outside of the pleural space and lie within the chest wall, a new ICD is required, and the old one should be removed. If the X-ray shows satisfactory positioning, inspect the drain tube for blockages. Normal saline (10-20 ml) can be used to flush the tube both away from the patient towards the drainage bottle and then towards the patient's chest separately via a three-way tap [3]. This maneuver can clear any blockage resulting from blood clots, fibrin, or debris. If this works, it is sensible to keep the drain on regular flushes (10-20 ml three or four times daily). If the drain remains non-functional, check if the dressing is too tight.

Problem 4

The drain is draining blood (hemothorax).

Reason: This can happen due to the rupture of the intercostal artery during drain insertion or as a complication of intrapleural fibrinolysis.

Solution: Immediate escalation to a senior physician is needed to ensure hemodynamic stability. Ensure intravenous access, administer intravenous fluids, and perform tests for a full blood count to check hemoglobin and hematocrit, along with blood grouping and cross-matching. Reverse any identified coagulopathy, and if on intrapleural fibrinolysis, stop it. Administer blood products as required. Once hemodynamic stability is achieved, perform imaging and consult the cardiothoracic team with imaging results on hand. You may need to speak with an interventional radiologist if necessary.

Problem 5

There is partial dislodgement of the chest drain tube.

Solution: Check if the side holes are still within the chest cavity. The tube can be re-sutured in its existing position. It should not be pushed back into the chest to avoid introducing the risk of infection. If the side holes are visible in the wound or outside the chest, remove the drain tube and replace it with a new one.

## Conclusions

It is ideal for every hospital to conduct small teaching sessions to train its junior doctors and other members of the medical on-call team on troubleshooting an ICD. Overnight, when the expertise of the respiratory team may not be available, adequate ICD troubleshooting skills can significantly help to manage adverse clinical situations. We have presented an example of how simple troubleshooting can save a patient from unnecessary and further invasive procedures. 
